# Travelers to U.S.: Zika Virus Knowledge, Attitudes, and Determinants of Practices in the Middle East—Insights for Future Awareness Campaigns

**DOI:** 10.3390/ijerph16142517

**Published:** 2019-07-14

**Authors:** Eman Y. Abu-rish, Eman R. Elayeh, Michael J. Browning

**Affiliations:** 1 Department of Biopharmaceutics and Clinical Pharmacy, School of Pharmacy, The University of Jordan, Amman 11942, Jordan; 2 Department of Immunology, Leicester Royal Infirmary, Leicester LE1 5WW, UK; 3 Department of Infection, Immunity and Inflammation, University of Leicester, Leicester LE1 9HN, UK

**Keywords:** *Aedes*, arboviruses, Middle East, traveler health, Zika virus

## Abstract

Travelers act as sentinels for the spread of Zika virus. Imported Zika cases and the presence of Zika virus-transmitting mosquitoes have been documented in the Middle East. However, data on travelers’ knowledge, attitude and practices regarding Zika and its prevention measures within the Middle East are scarce. This study aimed to address this issue in a sample of Jordanian and non-Jordanian travelers to U.S. in Jordan. A paper-based questionnaire was distributed to 301 travelers to U.S. in Queen Alia International airport, Amman, Jordan. Only 2.7% of the travelers knew that Zika is associated with birth defects. A total of 10.4% of the participants knew that the bite of infected mosquitoes is a route of Zika transmission. Only 12.6% of respondents correctly identified Zika prevention measures. The level of education and future plans for pregnancy were significantly associated with a high knowledge score (R^2^ = 0.140, *p*-value < 0.005). Although 76.2% of the travelers perceived Zika as a health threat, only 11.2% believed in the efficacy of the prevention measures. Formulation of educational campaigns within Middle Eastern countries and development of awareness strategies regarding Zika and its prevention within the airports are required. This is particularly essential with the upcoming 2022 FIFA World Cup in Qatar.

## 1. Introduction

Zika virus disease is an arboviral infection that is transmitted to humans predominantly through infected female *Aedes* species mosquitos. In particular, *Aedes aegypti* and *Aedes albopictus* are currently considered the main mosquito vectors of Zika virus [1]. Zika is also transmitted through sexual activity, blood transfusion, and vertically from mother to fetus [2,3]. Although the infection is usually asymptomatic, it is sometimes associated with neurological complications such as Guillain-Barré syndrome (GBS), and several poor pregnancy outcomes including congenital microcephaly and miscarriage [4]. The latest outbreak of Zika in Latin America and the Caribbean in 2015 has triggered the World Health Organization (WHO) in 2016 to declare Zika a public health emergency of international concern [5]. Travelers were considered key players in the 2015 Zika outbreak, which was hypothesized to include participants of the World Cup in 2014, or possibly earlier during the Confederations Cup in 2013 [4]. Since then, several reports of traveler-associated Zika importation have been documented in Australia, U.S., China, Europe and the Middle east [4,6,7,8,9]. In the U.S, The Pan American Health Organization (PAHO) has reported that the number of imported Zika cases from Zika-infected areas to U.S, as of January 2018, was 5335 compared to 227 local mosquito-borne transmissions [10]. In the U.S., 452 symptomatic Zika disease cases were reported during 2017, of which 437 cases were in travelers returning from affected areas, and seven cases of acquired Zika were through presumed local mosquito-borne transmission in Florida (*N* = 2) and Texas (*N* = 5). Thus, travelers significantly participated in the spread of Zika in the Americas [10,11]. A similar risk of Zika circulation might be possible during the upcoming 2022 FIFA world cup in the Middle Eastern country, Qatar [12].

Several Middle Eastern countries are now reported to have Zika-transmitting mosquitoes (*Aedes aegypti*) but without reported mosquito-borne Zika cases [10]. *A. albopictus* vectors of Zika virus were also reported in several Middle Eastern countries [13]. Although the CDC considered Jordan as a country with no mosquitoes (*A. aegypti)* that spread Zika [11], *A. albopictus* has recently been reported in Jordan [14]. *A. aegypti*, although not reported in Jordan, was detected in neighboring countries, such as Syria, Israel, and Lebanon, and there is a high predicted probability of its occurrence in Northern Jordan [13]. In addition, other *Aedes*-transmitted infections, such as chikungunya and dengue, were reported in other Middle Eastern countries, such as Egypt, Saudi Arabia, Yemen, and Oman [13].

Urbanization, globalization, and international mobility are the three major factors that have lately contributed in the overseas spread of epidemic arboviral diseases such as Zika [5]. The Middle East has experienced massive waves of these factors, not only due to its transcontinental location, but also due to the several intra-regional conflicts that occurred in the past few decades [14]. In Jordan, a Middle Eastern country, these factors created a dramatic urge of the Jordanian population to target the U.S. for migration, educational and professional purposes. Furthermore, Jordan receives immigrants and refugees from other Middle Eastern countries [15], many of whom ultimately seek migration to the U.S. and other western countries [16]. These travelers could participate in the circulation of Zika, due to their possible poor knowledge and adherence to Zika prevention measures. Currently, prevention is the sole way of limiting the spread of Zika virus, owing to the lack of an FDA-approved drug or vaccine against this virus [17]. Therefore, it is critical to identify travelers’ gaps of knowledge, attitudes, and their practices towards Zika and its prevention measures, and to identify the factors associated with better adherence to the prevention measures.

To date, only one report has addressed this issue among travelers in the Americas [18]. Yet, knowledge and practices of travelers to U.S arriving from areas outside the Americas, particularly from the Middle East, have not been specifically addressed before. We have recently reported poor levels of Zika knowledge in the general population in Jordan [19]. Travelers act as the key players for the spread of Zika. Therefore, evaluation of travelers’ level of knowledge and practices of the prevention measures are of even higher international concern for the assessment of the role of the Middle East in Zika circulation. The main aim of the current study was to assess Zika knowledge and prevention measures of travelers to U.S.. This gives insights into the role of travelers in the spread of Zika while staying in the U.S., through their possible poor knowledge of the routes of transmission and poor adherence to prevention measures, where they might get infected and contribute to the spread of the virus. In addition, the current study represents knowledge of travelers in the Middle East, where the next FIFA world cup is going to be held, therefore, providing insights into the possible roles of travelers in the Middle East in regional Zika circulation. The results of this study provide implications for health-care policy makers, within the Middle East and the U.S., to formulate health strategies that specifically address travelers’ knowledge and practices towards Zika, before leaving their countries in the Middle East or as they arrive in the U.S. and, therefore, in restricting the role of travelers in Zika circulation. This could help in reducing the emergence and the spread of new variants of Zika virus.

## 2. Methods

### 2.1. Study Subjects

Study subjects were recruited from Queen Alia International Airport in Amman, the capital of Jordan. A sample of Jordanian and non-Jordanian travelers to U.S. (age ≥ 18 year) who provided verbal consent were considered eligible. Travelers in flights departing to the following airports in the U.S. were approached: Detroit Metro Airport, Michigan; John F. Kennedy International Airport, New York city; and Chicago O’Hare International Airport, Chicago. Subjects available in the airport waiting halls at the time of data collection were invited to participate in the survey. U.S. flights were chosen to recruit the sample population of this study, as in Queen Alia International airport—the largest international airport in Jordan—there were no direct flights to Central and South America where the risk of Zika was highest. Travelers to those areas had to go to U.S. prior to making connecting flights to Central and South America. Travelers to U.S. were chosen to participate, as the main aim of the study was to assess the role of travelers from the Middle East in Zika circulation in U.S. through their possible poor knowledge and adherence to Zika prevention measures.

### 2.2. Study Design and Data Collection

This cross-sectional study was carried out over the period from June to October 2017. Consenting travelers were enrolled in a 5–10 minute semi-structured interview with a research assistant who was available at least three hours before flight departure. The study was conducted in accordance with the Declaration of Helsinki and the protocol was approved by the Institutional Review Board at the University of Jordan hospital (IRB no. 215/2016).

The questionnaire was built after reviewing related literature [17,20]. Content and face validity were implemented by other colleagues in the field. The questionnaire was modified after piloting to a sample of 20 adults.

The questionnaire used in this study was a shorter version of our previously published questionnaire [19], containing 19 knowledge questions rather than the original 32 questions. The final questionnaire included more travel-relevant sociodemographic characteristics and knowledge questions. The questionnaire included the following four sections [19,21]: demographics and general characteristics of the participants; participants’ knowledge about Zika, mode of transmission, and prevention measures; participants’ attitude towards Zika and its prevention measures; and participants’ adherence to Zika prevention measures during staying in or returning from areas with Zika outbreaks (such as Florida, Texas, and Central and South America). The factors that would affect the adherence of travelers to Zika prevention measures were also assessed. Participants were asked to select as many factors as applied to them.

### 2.3. Statistical Analysis

Statistical analysis was carried out using SPSS version 20.0 (SPSS Inc., Chicago, IL, USA). Categorical variables were described using descriptive statistics including percentages and frequencies, while mean and standard deviation (SD) were used to describe continuous variables. A 19-item knowledge score was calculated by assigning either a score of 1 for correct answers or a score of 0 for incorrect or “I do not know” answers Participants having a knowledge score higher than or equal to the mean knowledge score were considered knowledgeable. Bivariate analysis was used to test the differences among the variables that affect the knowledge score. Parametric tests including independent sample t-test and one-way ANOVA were used if conditions of normality and equality of variances were met. Backward-stepwise multivariate linear regression analysis was then used to determine the strength of association of the factors that were significantly associated with high knowledge score in the bivariate analysis. All hypothesis testings were two-sided. A *p*-value of < 0.05 was considered significant.

## 3. Results

### 3.1. General Characteristics of Participants

A total of 500 participants were approached, of whom 301 consented to participate (60% response rate). The mean age of participants was 35.6 ± 7.4 (20–74) years. Participants’ sociodemographic characteristics are summarized in Table 1 (the first two columns). Most of the participants were going to visit the U.S. for tourism, medical treatment, or for visiting their relatives (95.6%, *n* = 280). None of the travelers surveyed had ever travelled to an area with a Zika outbreak. Less than half of the participants were Jordanian while the rest were from 10 other Middle Eastern countries, one African, and one European country. A very low proportion of the participants lived in America (2.0%, *n* = 6), worked in the medical field (1.2%, *n* = 3), had an income ≤ 500 Jordanian dinar ($700 USD) (0.7%, *n* = 2), were pregnant females (1.0%, *n* = 2), or were males with pregnant wives (3.4%, *n* = 6).

### 3.2. Knowledge about Zika infection

The mean knowledge score of respondents was 9.0 ± 2.9 (out of 19) and only 58.1% (*n* = 175) of respondents had a score higher than or equal to the mean knowledge score and, thus, were considered knowledgeable. The maximum score achieved was 15 out of 19 (2.7%, *n* = 8). Only 5.3% (*n* =16) of the participants knew the definition of Zika infection, its symptoms and the highest risk group (the three questions together). Most importantly, only about a quarter of the study population (24.1%, *n* = 72) knew that pregnant women are the highest risk group of Zika infection (Table 2).

Regarding Zika complications, only 0.3% (*n* = 4) of the population correctly responded to the three questions. Most of the study population incorrectly considered severe disease and death as common compilations of Zika infection (99.7%, *n* = 300), while only 2.7% (*n* = 8) knew that Zika infection during pregnancy can cause severe birth defects.

Similarly, only 1.7% (*n* = 5) of participants were able to correctly respond to the questions related to the modes of transmission. In particular, 89.6% of the respondents could not identify the bite of infected mosquitoes as the major route of Zika transmission. About 66.6% (*n* = 199) appropriately recognized that direct contact between individuals is not a mode of Zika transmission. With regards to Zika prevention measures, more than 50% of participants were aware of the prevention measures except for the need of using insect repellents for two weeks after returning from an affected area (25.6%, *n* = 76). Overall, only 12.6% (*n* = 38) of respondents correctly identified all of the prevention measures (eight questions together).

The age, the level of education, employment, and planning for pregnancy were associated with a significantly higher knowledge score (Table 1). Of note, the sociodemographic characteristics which accounted for very small proportions of the participants, as described under "General characteristics of participants", were not used for knowledge score calculation, as a valid comparison of the knowledge score of these subcategories with the rest of the study population cannot be performed.

### 3.3. Multivariate Linear Regression Model for the Factors Associated with High Knowledge Score

Factors that were found to have significant association with high knowledge score using bivariate analysis (Table 1) were further analyzed through multivariate linear regression analysis (backward method). The level of education and future plans for pregnancy were the only sociodemographic factors that were associated with a significantly higher level of knowledge (R^2^ = 0.140, *p*-value < 0.005) (Table 3).

### 3.4. Sources of Information about Zika Infection

**Figure 1:**
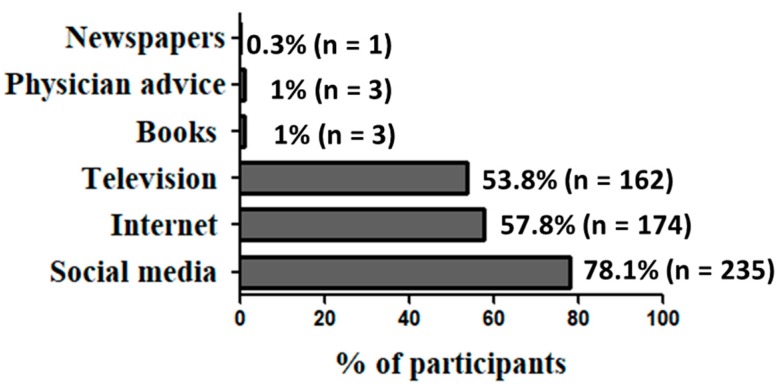
Sources of information about Zika. Participants were asked to choose as many sources that apply to them. Valid percentages are presented.

The most common sources of participants’ information about Zika were social media, followed by internet and television. On the other hand, much lower proportions of respondents relied on books, physician advice, or newspapers as sources of information (Figure 1).

### 3.5. Attitude of Participants towards Zika and Its Prevention Measures

Our results showed that only 11.2% (*n* = 33) of the participants believed in the efficacy of Zika prevention measures, despite 76.2% (*n* = 224) of them perceiving Zika as a health risk.

### 3.6. Factors Associated with Population Adherence to Zika Prevention Measures

**Figure 2:**
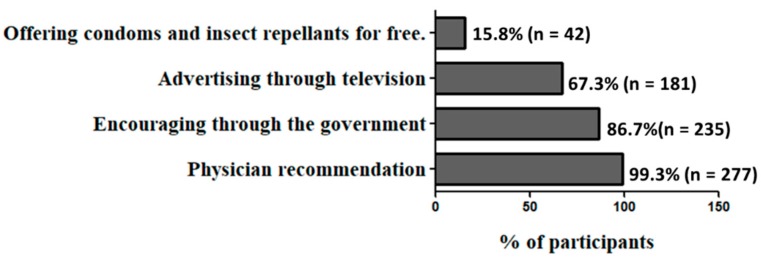
Factors that encourage participants to adhere to Zika prevention measures. Participants were asked to choose as many factors that apply to them. Valid percentages are presented.

Physician advice was the primary factor that encourages participants to adhere to Zika prevention measures (Figure 2), followed by the government role and television. However, the majority of participants (99.3%, *n* = 276) reported that they had never been advised about Zika by a physician. The least important factor was the free offering of condoms and insect repellants.

## 4. Discussion

This is the first study conducted in the Middle East to assess Middle East-to-Americas travelers’ Zika knowledge, attitude and beliefs. We revealed critical gaps in travelers’ knowledge regarding Zika definition, complications and its routes of transmission. The level of education and planning for future pregnancy were associated with a higher knowledge score. Most of the travelers perceived Zika as a health threat, yet they did not believe in the efficacy of the prevention measures. Physician recommendation was the most important factor in travelers’ willingness to adhere to the prevention measures, but only 1% reported having received advice on Zika from a physician.

Although Zika spread into the Americas was believed to be travel-associated [5], and despite of the global media coverage regarding Zika at the time of data collection, several gaps of knowledge were identified in the surveyed travelers. We showed that less than a quarter of the respondents knew that pregnant women are the highest risk group for this infection, less than 3% knew that Zika is associated with severe birth defects, and only 1 participant knew that severe infection and death due to Zika are uncommon complications of this virus. In contrast, 47% of pregnant women surveyed in Greece and 95% of the participants surveyed in New York City reported an association between Zika and microcephaly [22,23]. In our study, only 10.4% of participants identified the bite of infected mosquitoes as the major route of Zika transmission, compared with 80% of U.S. travelers and 85% of the survey participants in New York City [18,23]. Notably, travelers in this study have much lower level of knowledge regarding Zika complications and the major route of its transmission compared to participants in U.S. surveys. This might be attributed to the majority of the respondents in our study coming from the Middle East countries, and only 2% of them were actually living in the U.S. where the risk of Zika is higher than in the Middle East.

Considering the route of transmission, only 55.9% of respondents in this study knew of the sexual route of transmission. This was in parallel with previous surveys in U.S. where less than 50% of U.S. travelers in at risk states, 77% of travelers in Puerto Rico, and 59% of the participants in New York City knew this route of transmission [18,23]. This low level of knowledge of participants in this regard might increase the potential of traveler-mediated sexual transmission of Zika. Of note, according to the PAHO-WHO Zika Cumulative Case report in January 2018, 48 cases of Zika were acquired through sexual activity in the U.S. [10].

Only 12.6% of travelers correctly identified all of the prevention measures described in this study. Specifically, only 25.5% of respondents knew about using insect repellents for two weeks after returning from a Zika affected area. Knowledge about this prevention measure was not addressed before by other surveys. Additionally, only 59.8% of respondents in this study knew that male partners should use condoms (or other barriers) to reduce the chance of getting Zika from sex. This proportion was also lower than previously reported in New York City (74%) [23].

Multivariate linear regression analysis showed that having a Bachelor degree or higher level of education was associated with a higher level of knowledge. This was consistent with previous reports which surveyed pregnant women in Greece or adults in American Samoa [22,24].

In New York City, pregnancy was associated with higher Zika knowledge [23]. In our study, pregnant women accounted for only 1% of the study population, therefore, valid associations regarding this subgroup could not be established. However, we found that planning for future pregnancy was associated with a higher knowledge score. This might be ascribed to the fact that Zika is associated with poor pregnancy outcomes and, thus, those planning for pregnancy had more concerns and greater awareness regarding this disease.

In this study, participants were travelers from 13 different nationalities, while in our previous report in Jordan, participants were non-travelling Jordanians [19]. In the current report, there was no statistical difference in the knowledge score between Jordanian and non-Jordanian. Further, differences in knowledge scores among the different nationalities could not be established due to the limited sample size of each subgroup.

Social media, followed by internet and television, were the most commonly used sources of information by the participants in this study, while only 1% of them relied on either books or physician advice. Reliance on health-care providers as sources of information was also low in Qatar (1.1%) and U.S. (9%) [23,25].

Three-quarters of the respondents perceived Zika as a health threat, however, only 11.2% of them believed in the efficacy of the prevention measures. This attitude might be attributed to the fact that almost all of the participants in this study have not been advised regarding Zika by a physician before. Thus, the role of health-care providers in this regard should be encouraged, particularly as we showed that the major determinant of participants’ intention to practice Zika prevention measure was physician recommendation. Additionally, health-care providers are the most trusted sources of medical information [26]. Other determinants of participants’ intention to adhere to prevention measures were the role of government and television, while the least important factor was the free offering of condoms and insect repellants (15.8%). Similarly, only 21% of pregnant immigrants in U.S. reported the cost of insect repellent to be problematic [27].

Noteworthy, none of the travelers surveyed in this study have ever travelled to an area with Zika outbreak, therefore, assessment of their practice of Zika prevention measures could not be performed.

## 5. Limitations

In this study, the proportion of participants travelling to Latin and south America, where the risk of Zika is high, could not be determined as none of them answered the question related to their final destination in the Americas. Therefore, our results should be interpreted cautiously, as the data were extracted from travelers to U.S. and, therefore, cannot be extrapolated to represent travelers to countries in Latin and South America.

## 6. Conclusions

The findings of this study revealed that travelers to U.S. through a Middle Eastern airport had poor knowledge regarding critical aspects of Zika disease. This included poor knowledge of the major routes of its transmission (bites of infected mosquitoes and sexual transmission) and its prevention measures (continuous use of insect repellent for two weeks after returning to areas with low risk of Zika, and using condoms). In addition, most of participants have negative attitudes regarding the efficacy of the prevention measures. In view of this, such travelers might significantly participate in the transmission of Zika in U.S. Communication with health-care providers and government information are the most important determinants of intention to practice prevention measures. Action plans should take place within the Middle East airports to establish educational and awareness strategies in order to enhance travelers’ knowledge and practices towards Zika and its prevention measures.

## Figures and Tables

**Table 1:** Sociodemographic characteristics of all participants (*n* = 301) and their association with the knowledge scores.

Variable	(%)^1^ n	Knowledge score	*p*-value
		Mean ± SD	
**Age *^2^***			
<35	48.1 (143)	8.6 ± 3.2	0.004 *
≥35	51.9 (154)	9.6 ± 2.4	
**Gender *^2^***			
Females	32.2 (97)	8.7 ± 2.8	0.183
Males	67.8 (204)	9.2 ± 3.1	
**Marital status *^3^***			
Married	89.0 (266)	9.1 ± 2.9	0.425
Single	7.4 (22)	8.2 ± 3.4	
Others ^4^	3.6 (11)	8.9 ± 3.4	
**Education *^2^***			
Less than bachelor degree	16.7 (50)	7.5 ± 3.4	0.001*
Bachelor degree or higher	83.3 (251)	9.0 ± 2.7	
**Employment *^2^***			
Not working	18.9 (57)	8 ± 3.5	0.019*
Working full time or part time	81.1 (244)	9.2 ± 2.7	
**Having children *^2^***			
Yes	71.3 (211)	9.0 ± 2.8	0.835
No	28.7 (85)	8.9 ± 3.2	
**Future plans of the partners for pregnancy *^2^***			
Yes	46.5 (140)	9.6 ± 2.7	0.001 *
No	53.5 (161)	8.5 ± 3.0	
**First trip to America ^2^**			
Yes	10.0 (30)	8.3 ± 3.0	0.105
No	90.0 (269)	9.2 ± 2.8	
**Visiting America for different purposes (tourism, medical treatment…etc) *^2^***			
Yes	95.6 (274)	9.0 ± 2.9	0.873
No	4.4 (12)	9.2 ± 2.6	
**Nationality *^2^***			
**Jordanian**	48.7 (134)	9.0 ± 2.8 8.8 ± 3.0	0.57
**Non-Jordanian**	51.3 (142)		
Palestinian	5.8 (16)		
Saudi	6.2 (17)		
Syrian	5.1 (14)		
Egyptian	8.7 (24)		
Lebanese	3.6 (10)		
Iraqi	8.0 (22)		
Kuwaiti	4.0 (11)		
Swedish	4.0 (11)		
Yemeni	2.2 (6)		
Omani	2.2 (6)		
Qatari	1.1 (3)		
Tunisian	0.4 (1)		

^1^ Valid percent; ^2^ Independent sample t-test; ^3^ One way ANOVA; ^4^ Widow, divorced; * Significant at *p*-value < 0.05.

**Table 2:** Participants’ knowledge about Zika, its mode of transmission, and its prevention measures.

Question	Correctly answered % (*n*)
**Definition, signs and symptoms, risk groups, and complications**	
Zika is a disease caused by a virus that is primarily spread to people through the bite of an infected mosquito.	7.7 (23)
The most common signs and symptoms are fever, rash, joint pain, and conjunctivitis (red eyes).	79.6 (238)
The highest risk group is pregnant women.	24.1 (72)
**Complications of Zika infection**	
Zika infection during pregnancy can cause severe birth defects, including microcephaly.	2.7 (8)
Severe disease requiring hospitalization due to Zika is common.*	0.3 (1)
Death from Zika infection is common.*	0.3 (1)
**Mode of transmission**	
**Zika is transmitted primarily through the bite of infected mosquitoes**.	10.4 (31)
Sexual contact.	55.9 (167)
Vertically from a pregnant woman to her fetus.	81.3 (243)
Through blood transfusion.	96.0 (287)
Directly from one person to another through casual contact.*	66.6 (199)
From a mother to fetus through breastfeeding.*	17.1 (51)
**Prevention measures**	
**Prevention of mosquito bites**	
**The use of insect repellents is necessary to prevent mosquito’s bite**.	71.9 (215)
Wearing long-sleeved shirts and long pants is not necessary. *	69.2 (207)
Stay in places with air conditioning or window and door screens.	76.3 (228)
Removing standing water and rubbish around your home.	75.3 (225)
Asymptomatically infected individuals returning from affected areas to non-affected areas should continue the use of insect repellents for a minimum of extra 14 days.	25.6 (76)
**Prevention of transmission through sex**	
Male partners should use condoms (or other barriers) to reduce the chance of getting Zika from sex.	59.8 (177)
**Prevention of transmission during pregnancy**	
Pregnant women should not travel to areas with Zika.	68.6 (201)

* The correct answer for the question is “NO”.

**Table 3:** Results of linear regression analysis for factors associated with high knowledge score.

Variables	SE	B ^1^	*T*	*p*-value
Constant	3.444	-	3.702	<0.005 *
Age	0.345	0.114	1.936	0.054
Level of education	0.472	0.202	3.393	0.001 *
Employment	0.225	0.078	1.281	0.201
Planning for pregnancy	0.336	−0.122	−0.180	0.002 *

^1^ Standardized coefficient; * Significant at *p*-value < 0.05.
